# The EuroQol-5D questionnaires: a brief outline

**DOI:** 10.1093/occmed/kqaf130

**Published:** 2025-12-24

**Authors:** Matteo Carpi, Flora Gatti, Ciro De Vincenzo

**Affiliations:** Department of Human Neuroscience, Sapienza University of Rome, Rome, 00185, Italy; Department of Social Sciences, University of Naples Federico II, Naples, 80138, Italy; Department of Philosophy, Sociology, Education and Applied Psychology, University of Padua, Padua, 35131, Italy

## Brief history

The EuroQol-5D (EQ-5D) is a standardised instrument for measuring generic health-related quality of life, developed by the EuroQol Group in the late 1980s. The first version of the ‘EuroQol instrument’, featuring three levels of severity per dimension (now referred to as EQ-5D-3L), was introduced in 1990 [1] and later formally named EQ-5D. Since 2009, the EQ-5D family has expanded to include three versions: the original three-level EQ-5D-3L, an enhanced five-level version (EQ-5D-5L), and a youth version (EQ-5D-Y) designed for children and adolescents [2]. The 5 L version, officially released in 2011, was developed to improve sensitivity and reduce the limitations observed in the 3 L version, notably its coarse scaling—that is, the restricted number of response levels, which constrain response options, and may reduce discriminatory power.

Since its introduction, the EQ-5D has seen widespread adoption in both research and clinical practice. It is now one of the most commonly used generic quality-of-life measures globally, with applications in clinical trials, population health surveys, and health economic evaluations. By 2015, over 17 000 studies had been registered with the EuroQol Group using the EQ-5D across more than 80 clinical areas and 40 different programmes and settings [3]. As of October 2025, over 16 000 documents indexed in MEDLINE/PubMed directly refer to the EQ-5D, placing it among the most widely used patient-reported outcome measures (PROMs). The instrument’s reach is global: both the EQ-5D-3L and the newer EQ-5D-5L have been translated into over 150 languages and are available in various modes of administration, including self-report and interviewer-administered formats.

## Description

The EQ-5D questionnaire is designed to be a simple, self-administered measure of current health status. It comprises five dimensions of health: mobility, self-care, usual activities (i.e. ability to engage in everyday tasks), pain/discomfort, and anxiety/depression. Each dimension is assessed with a single item, and respondents indicate their level of problem severity on the day of assessment. The original EQ-5D-3L form includes three response levels per dimension: no problems, some problems, or extreme problems (e.g. ranging from ‘no pain or discomfort’ to ‘extreme pain or discomfort’ in the pain dimension). The expanded EQ-5D-5L version offers a more nuanced five-level response scale: no problems, slight, moderate, severe, and extreme problems (or unable to do for certain activities) [4]. These intermediate levels enable respondents to provide a more accurate and sensitive account of their health than the older 3 L scale.

A respondent’s answers yield a five-digit health profile (one digit per dimension). For example, a health state of ‘11111’ indicates no problems in any domain; essentially perfect health, whereas ‘23545’ would reflect various moderate and severe issues across dimensions. Hence, the 3 L descriptive system defines 243 possible health states (3^5^ combinations), while the 5 L system defines 3125 health states (5^5^ combinations). In both versions, the EQ-5D includes a visual analogue scale (EQ-VAS): a 20-cm vertical scale on which respondents rate their overall health from 0 (worst imaginable health) to 100 (best imaginable health). The EQ-VAS yields an immediate, self-rated quantitative measure of health status, complementary to the descriptive profile [5].

In addition to the descriptive summary and EQ-VAS score, each EQ-5D health state can be converted into a single summary index value (or utility weight), derived from preference-elicitation studies conducted in representative general-population samples. As a result of this weighting, health states are typically valued on a scale anchored at 1 (full health) and 0 (equivalent to being dead), and some states may receive negative values, conventionally termed states worse than dead (SWD), reflecting aggregated valuations rather than individual patient judgements [6]. These index values are most commonly used in the calculation of quality-adjusted life years (QALYs), particularly in the context of health economic evaluation [7].

Notably, the scoring and analysis of EQ-5D data remains a subject of ongoing methodological debate. Updated guidance manuals are available to support researchers and practitioners from data collection through to purpose-specific analysis [8], and a comprehensive library of documentation and resources is accessible on the dedicated EuroQol website [9].

Thanks to its brevity (a two-page form including the EQ-VAS) and ease of use, the EQ-5D can be completed in just a few minutes and is feasible across a range of settings, making it a practical choice for monitoring health outcomes in both clinical care and large-scale public health research.

## Validity

The EQ-5D has been extensively tested for reliability and validity across diverse populations and languages. Given the instrument’s aim of capturing five distinct health dimensions, test–retest reliability has typically been prioritised over internal consistency as the principal indicator of reliability, and studies have reported intra-class correlation coefficients for repeated administrations that generally indicate good temporal stability [10, 11]. Furthermore, both the EQ-5D-3L and EQ-5D-5L demonstrate fair convergent validity, with domain scores and index values correlating in the expected directions with other multi-domain or unidimensional measures of physical and emotional health status [11, 12].

To address the sensitivity limitations observed in the original three-level EQ-5D (especially in healthier or milder conditions), the psychometric properties of the 5 L version have been systematically investigated, and findings show that increasing the number of response levels to five significantly improves discriminatory power, while preserving feasibility [10, 11]. Indeed, the 5 L version is more effective than the 3 L at detecting small or moderate health changes that might otherwise be missed, and these enhancements come without sacrificing the instrument’s brevity or ease of use. Nonetheless, a degree of ceiling effect may still be present in general population samples [13], for which a measure of health deviation (i.e. deterioration from full health) may naturally yield skewed results towards the upper end. This tendency contrasts with instruments that capture positive health attributes (e.g. physical fitness), where scoring distributions may differ. Despite this, the available evidence supports the EQ-5D, and particularly the 5 L version, as a reliable and valid tool for assessing health-related quality of life across a wide range of contexts.

## Key research and applications

Since its release, the EQ-5D has been applied in a vast array of studies and has become a standard instrument for health outcomes research. As previously noted, published studies span more than 80 health conditions and intervention areas, ranging from surgical outcomes and chronic disease management to mental health and primary care. The EQ-5D’s widespread use is due in large part to its utility in health economics, with many health technology assessment agencies, including the National Institute for Health and Care Excellence (NICE) in the UK, recommending EQ-5D as a preferred tool for generating QALYs in cost–utility analyses [14]. Numerous countries have established population norm values using EQ-5D, which are used to inform public health policy and guide resource allocation [9]. Additionally, the instrument’s concise profile of five dimensions enables researchers to identify which aspects of health are most affected in different groups [8].

Of particular relevance to occupational health research, the EQ-5D has been used to evaluate how work and workplace factors are associated with health-related quality of life. Its five domains map closely onto core aspects of functional capacity and general well-being, making it well suited to working populations. For example, a large Korean panel study of female shift workers (*n* = 2238) employed EQ-5D to assess the health impacts of non-traditional work schedules, highlighting the toll of both night work and rotating shifts on health [15]. Similarly, in a general working population sample from Japan (*n* = 1986), significant associations were found between work-related factors (such as job strain, effort-reward imbalance, and co-worker support) and QALYs derived from the EQ-5D [16].

More broadly, the EQ-5D’s ‘usual activities’ dimension is directly relevant to work ability, and its pain/discomfort and anxiety/depression scales capture symptoms commonly observed in occupational settings (e.g. musculoskeletal pain, work-related stress). By providing a simple yet robust summary of health status, the EQ-5D enables employers, clinicians, and policymakers to quantify the burden of occupational injuries or diseases, and to evaluate the cost-effectiveness of workplace health initiatives in a standardised manner.

## Accessing the instrument

Distribution and licensing for all EQ-5D versions are managed by the EuroQol Research Foundation, which helps ensure consistent standards for its global use. Validated translations, administration formats (including interviewer-rated and proxy versions), population norms, and user support materials are available via the Foundation’s official website [9].
